# Ginger-Derived 3HDT Exerts Antiproliferative Effects on Breast Cancer Cells by Apoptosis and DNA Damage

**DOI:** 10.3390/ijms24065741

**Published:** 2023-03-17

**Authors:** Chung-Yi Chen, Yan-Ning Chen, Jun-Ping Shiau, Jen-Yang Tang, Ming-Feng Hou, Hsueh-Wei Chang

**Affiliations:** 1 Department of Nutrition and Health Sciences, School of Medical and Health Sciences, Fooyin University, Kaohsiung 83102, Taiwan; 2 Graduate Institute of Medicine, College of Medicine, Kaohsiung Medical University, Kaohsiung 80708, Taiwan; 3 Division of Breast Oncology and Surgery, Department of Surgery, Kaohsiung Medical University Hospital, Kaohsiung Medical University, Kaohsiung 80708, Taiwan; 4 School of Post-Baccalaureate Medicine, Kaohsiung Medical University, Kaohsiung 80708, Taiwan; 5 Department of Radiation Oncology, Kaohsiung Medical University Hospital, Kaohsiung 80708, Taiwan; 6 Department of Biomedical Science and Environmental Biology, College of Life Science, Kaohsiung Medical University, Kaohsiung 80708, Taiwan; 7 Institute of Medical Science and Technology, National Sun Yat-sen University, Kaohsiung 80424, Taiwan; 8 Center for Cancer Research, Kaohsiung Medical University, Kaohsiung 80708, Taiwan

**Keywords:** ginger, TNBC, DNA damage, apoptosis, oxidative stress

## Abstract

Ginger-derived compounds are abundant sources of anticancer natural products. However, the anticancer effects of (*E*)-3-hydroxy-1-(4′-hydroxy-3′,5′-dimethoxyphenyl)-tetradecan-6-en-5-one (3HDT) have not been examined. This study aims to assess the antiproliferation ability of 3HDT on triple-negative breast cancer (TNBC) cells. 3HDT showed dose-responsive antiproliferation for TNBC cells (HCC1937 and Hs578T). Moreover, 3HDT exerted higher antiproliferation and apoptosis on TNBC cells than on normal cells (H184B5F5/M10). By examining reactive oxygen species, mitochondrial membrane potential, and glutathione, we found that 3HDT provided higher inductions for oxidative stress in TNBC cells compared with normal cells. Antiproliferation, oxidative stress, antioxidant signaling, and apoptosis were recovered by *N*-acetylcysteine, indicating that 3HDT preferentially induced oxidative-stress-mediated antiproliferation in TNBC cells but not in normal cells. Moreover, by examining γH2A histone family member X (γH2AX) and 8-hydroxy-2-deoxyguanosine, we found that 3HDT provided higher inductions for DNA damage, which was also reverted by *N*-acetylcysteine. In conclusion, 3HDT is an effective anticancer drug with preferential antiproliferation, oxidative stress, apoptosis, and DNA damage effects on TNBC cells.

## 1. Introduction

Breast cancer is the most prominent cancer with deadly outcomes in women [1]. Three primary biomarkers are known for differential expression in most breast cancer types, namely the estrogen receptor (ER), progesterone receptor (PR), and human epidermal growth factor receptor 2 (HER2) [2], and breast cancer shows different positive or negative expression combinations for these three markers. Only 10–15% of breast cancer patients are negative for ER/PR/HER2 [3], i.e., triple-negative breast cancer (TNBC). Non-TNBC cells are generally cured by specific target therapies to one of these three markers. For example, the HER2 antibody can specifically target and destroy HER2 to HER2-positive non-TNBC cells [4]. Hormone therapy is suitable for treating ER/PR-positive non-TNBC [5,6].

TNBC patients exhibit more early and frequent recurrence and poor survival than non-TNBC patients, which is attributed to the nature of TNBC cells, making it the most aggressive subtype of breast cancer [7,8], with a weak response to current target therapies for non-TNBC. TNBC is a status consisting of several heterogenous tumors [9]. Single chemotherapy fails to cure the majority of TNBC patients. Chemoresistance also reduces therapeutic effects on TNBC [10]. New drug treatments for TNBC are more urgently needed than non-TNBC. Moreover, clinical drugs are frequently associated with side effects for breast cancer patients. Accordingly, it is essential to continue identifying more anticancer drugs to cure TNBC cells without common side effects.

Ginger (*Zingiber officinale* Roscoe) provides a herbal seasoning spice and has long been commonly used in herbal medicines. Ginger has functions for treating nausea and muscle discomfort, toothache, and pain [11,12]. Crude extracts of ginger show anticancer effects [13]; therefore, this warrants a detailed assessment of the bioactive compounds in ginger extracts. Recently, several compounds were identified from the rhizomes of ginger extracts, including gingerols, shogaols, gingediols, zingerone, dehydrozingerone, gingerinone, and diarylheptanoids, which function for the anti-inflammation and antiproliferation of cancer [14,15,16,17]. For example, 6-shogaol and 10-shogaol suppress the proliferation of prostate cancer cells [15], and 10-gingerol inhibits the metastasis effects of TNBC in vivo [17].

Moreover, several ginger-derived compounds exhibit an oxidative-stress-generating ability for killing cancer cells. For example, gingerenone A inhibits the proliferation of breast cancer cells by upregulating oxidative stress [18], and 6-gingerol triggers oxidative stress and inhibits the proliferation of gastric cancer cells [19]. However, several ginger-derived compounds have not been fully investigated regarding their medicinal effects.

(*E*)-3-hydroxy-1-(4′-hydroxy-3′,5′-dimethoxyphenyl)-tetradecan-6-en-5-one [20], namely 3HDT, is a novel phenylalkanoid compound isolated from the rhizomes of *Z. officinale*; however, this new compound was described without providing detailed biological information. The present investigation aims to evaluate the antiproliferation impacts of 3HDT on TNBC cells. The detailed anticancer mechanism of 3HDT is also examined using TNBC and normal breast cells.

## 2. Results

### 2.1. Cell Viability of 3HDT-Treated TNBC Cells

**Figure 1:**
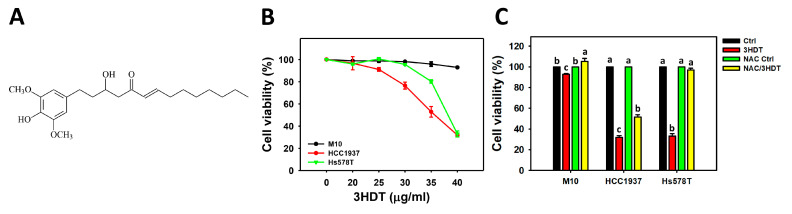
Cell viabilities of 3HDT treatment. (**A**,**B**) Structure and CCK-8 assay of 3HDT for 24 h. (**C**) CCK-8 assay of *N*-acetylcysteine (NAC)/3HDT. 3HDT and NAC/3HDT indicate 24 h 3HDT treatment (40 μg/mL) without and with NAC pretreatment, respectively. 3HDT was dissolved in DMSO. The DMSO concentration of different 3HDT treatments was adjusted to the same (0.1%). Control (Ctrl) indicates the vehicle control containing 0.1% DMSO. NAC control (NAC Ctrl) indicates the NAC/0.1% DMSO. Data: mean ± SD (*n* = 3). Treatments differ significantly for multiple comparisons when the lower-case letters are not overlapping (*p* < 0.05). Different treatments of the same cell line were compared for statistical analysis. In the example of M10 cells, NAC/3HDT, NAC control, and 3HDT are assigned with “a, b, c” by the JMP software, indicating significant differences between each other because the letters are non-overlapped. In contrast, control and NAC control show the same letter “b”, indicating a non-significant difference.

Following 3HDT (Figure 1A) treatment, the cell viabilities (%) at 24 h for the CCK-8 assay of TNBC cells (Hs578T and HCC1937) were decreased; however, they showed higher viabilities for normal breast cells (M10) than TNBC cells (Figure 1B). The IC_50_ values of 3HDT for Hs578T and HCC1937 cells were 38.22 ± 0.23 and 35.67 ± 0.95 μg/mL, respectively.

Moreover, NAC/3HDT, the reactive oxygen species (ROS) inhibitor *N*-acetylcysteine (NAC) pretreatment/3HDT post-treatment, showed higher viability than 3HDT treatment of TNBC cells (Figure 1C), suggesting that 3HDT promotes preferential antiproliferation of TNBC cells via oxidative stress generation.

### 2.2. Cell Cycle Distribution of 3HDT-Treated TNBC Cells

**Figure 2:**
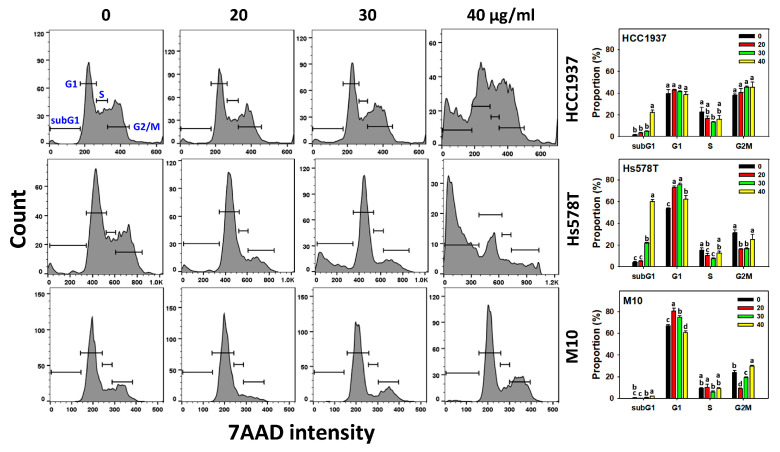
Cell cycle changes for 3HDT treatment. Cell cycle flow cytometry assay of 3HDT (control, 20, 30, and 40 μg/mL) for 24 h was performed. Data: mean ± SD (*n* = 3). Treatments differ significantly for multiple comparisons when the letters are not overlapping (*p* < 0.05).

The dysregulation of cell cycle progression inhibits cancer cell proliferation [21]. After checking with the 7AAD assay, the impact of cell cycle modulation by 3HDT was examined (Figure 2). Following the 3HDT treatment, subG1 (%) was higher in TNBC cells than in normal cells. Except for the subG1 proportion, the G1, S, and G2/M cell phases showed a nonsignificant difference for HCC1937 cells. The primary cell cycle change in Hs578T is the increment of subG1, accompanied by G1 arrest.

For normal M10 cells, G1 was mildly increased for 20 and 30 μg/mL but decreased in 40 μg/mL 3HDT without subG1 increment. In contrast, G2/M was decreased in 20 and 30 μg/mL 3HDT but slightly increased in 40 μg/mL in M10 cells.

These results suggest that 3HDT promotes preferential subG1 accumulation (an apoptosis-like indicator) of TNBC cells but not in normal cells, while 3HDT differentially modulated cell cycle arrest for TNBC and normal cells.

### 2.3. Apoptosis of 3HDT-Treated TNBC Cells

**Figure 3:**
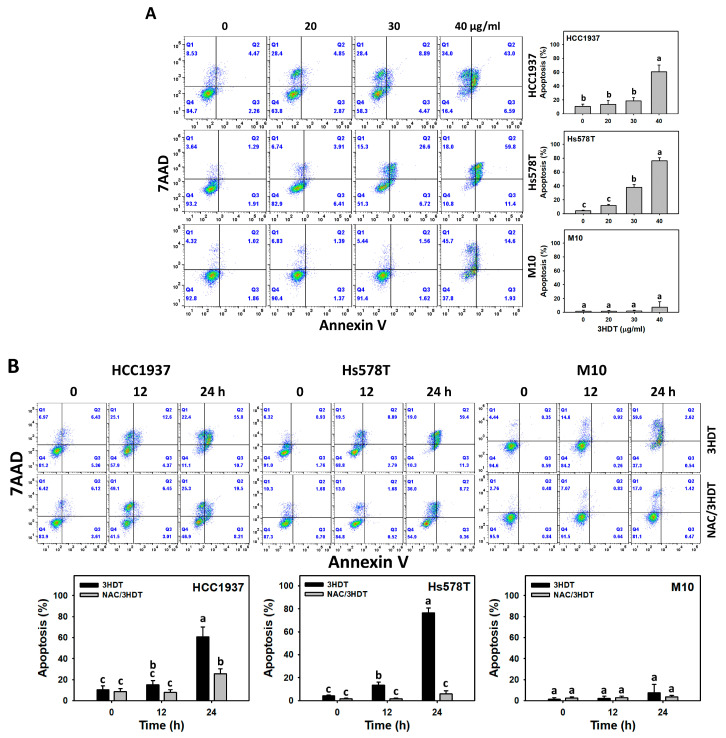
Annexin V status of 3HDT treatment. (**A**) Annexin V flow cytometry assay of 3HDT (control, 20, 30, and 40 μg/mL) for 24 h. (**B**) Annexin V flow cytometry assay of NAC/3HDT. 3HDT and NAC/3HDT indicate 12 or 24 h 3HDT treatment (40 μg/mL) without and with NAC pretreatment, respectively. Q1 is classified as necrosis region [23,24]. Q2 and Q3: Annexin V (+)/7AAD (+/−) (%) is regarded as apoptosis (%) [23,24]. Data: mean ± SD (*n* = 3). Treatments differ significantly for multiple comparisons when the letters are not overlapping (*p* < 0.05).

Annexin V can bind the phosphatidylserine in the outer plasma membrane of apoptotic cells [22]. Annexin V-FITC was applied to detect the apoptosis status. Following 3HDT treatment, the annexin-V-assessed apoptosis was higher in TNBC cells in a dose-dependent manner than in normal cells (Figure 3A). Although M10 showed a low proportion of apoptosis, the putative necrosis proportion was higher than in TNBC cells.

Similarly, 3HDT treatment also showed the upregulation of annexin-V-detected apoptosis in time-dependent manners for TNBC cells rather than in normal cells (Figure 3B). This indicates that 3HDT preferentially induces apoptosis in TNBC but not normal cells. Moreover, 3HDT showed a higher annexin V (+) level than NAC/3HDT treatment of TNBC cells, suggesting that 3HDT promotes the preferential apoptosis (annexin V) of TNBC cells via oxidative stress generation.

### 2.4. Caspase 3/8/9 Signaling of 3HDT-Treated TNBC Cells 

**Figure 4:**
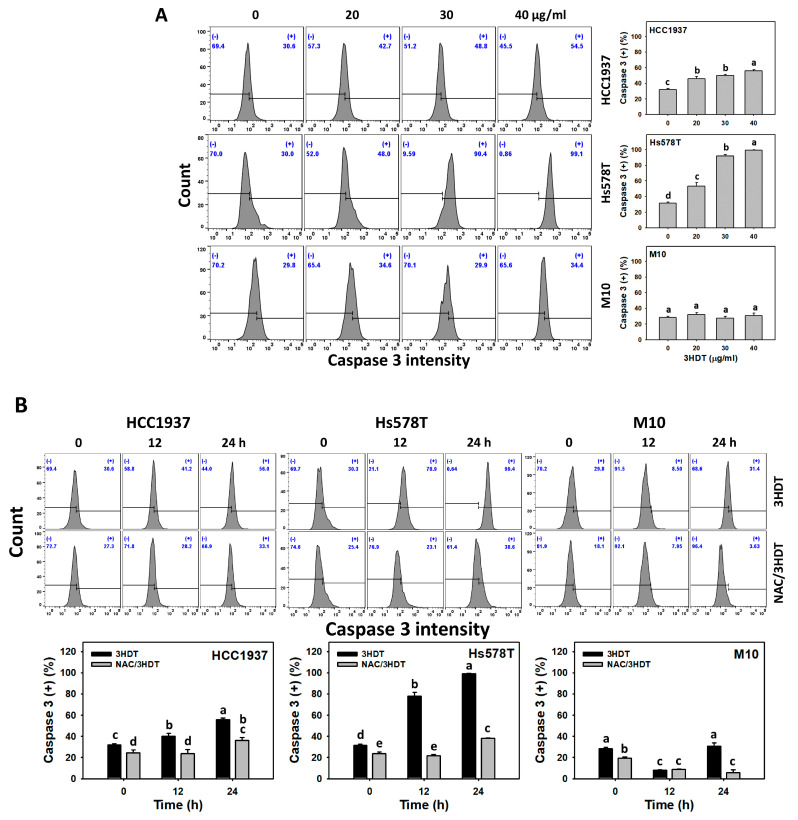

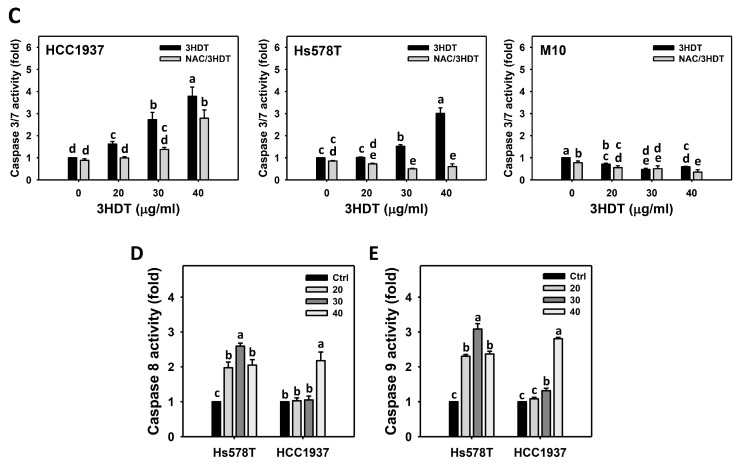
Caspase 3/8/9 status of 3HDT treatment. (**A**) Caspase 3 flow cytometry assay of 3HDT (control, 20, 30, and 40 μg/mL) for 24 h. (+) is marked as caspase 3 (+), which indicates the proportion with high caspase 3 level, while (−) indicates the proportion with low caspase 3 level. (**B**) Caspase 3 flow cytometry assay of NAC/3HDT. 3HDT and NAC/3HDT indicate 12 or 24 h 3HDT treatment (40 μg/mL) without and with NAC pretreatment, respectively. (**C**) Caspase 3/7 luminescence assay of NAC/3HDT for 24 h. 3HDT (control, 20, 30, and 40 μg/mL) was posttreated after NAC pretreatment. (**D**,**E**) Caspase 8 and caspase 9 luminescence assay of 3HDT (control, 20, 30, and 40 μg/mL) for 24 h. Data: mean ± SD (*n* = 3). Treatments differ significantly for multiple comparisons when the letters are not overlapping (*p* < 0.05).

Caspase 3 is the apoptotic downstream executioner that interacts with the upstream extrinsic caspase 8 and intrinsic caspase 9 [25,26]. In general, caspase 3 was first examined to check the apoptosis signaling [27] before validating the involvement of caspases 8 and 9. Based on flow cytometry, the activated caspase 3 (+) level was higher in 3HDT-treated TNBC cells in a dose-dependent manner than in normal cells (Figure 4A). Similarly, the 3HDT treatment showed the upregulation of caspase 3 in time-dependent manners for TNBC cells rather than normal M10 cells (Figure 4B). 3HDT showed an increased activated-caspase 3 (+) level compared with NAC/3HDT treatments of TNBC cells (Figure 4B). These results suggest that 3HDT promotes the preferential activation of caspase 3 (apoptosis) in TNBC cells via oxidative stress generation.

To further confirm caspase 3 apoptosis signaling, caspase 3/7 activity was examined as they are the key modulators of apoptosis [28]. The caspase 3/7 luminescence assay showed that its apoptotic activation was higher in 3HDT-treated TNBC cells in a dose-dependent manner than in normal cells (Figure 4C). Moreover, 3HDT showed a higher caspase 3/7 level than NAC/3HDT treatment of TNBC cells, suggesting that 3HDT promotes the preferential caspase 3/7 activation (apoptosis) of TNBC cells via oxidative stress generation.

As caspase 3 activation was validated (Figure 4A–C), the upstream extrinsic (caspase 8) and intrinsic (caspase 9) signaling pathways were assessed (Figure 4D,E). All treatments of 3HDT showed a higher caspase 8 and 9 activity in Hs578T cells than in the control. Only 40 μg/mL and 30–40 μg/mL 3HDT showed higher caspase 8 and 9 activity in HCC1937 cells. These results suggest that 3HDT promotes caspase 8 and 9 (extrinsic and intrinsic apoptosis) in TNBC cells.

### 2.5. ROS Status of 3HDT-Treated TNBC Cells

**Figure 5:**
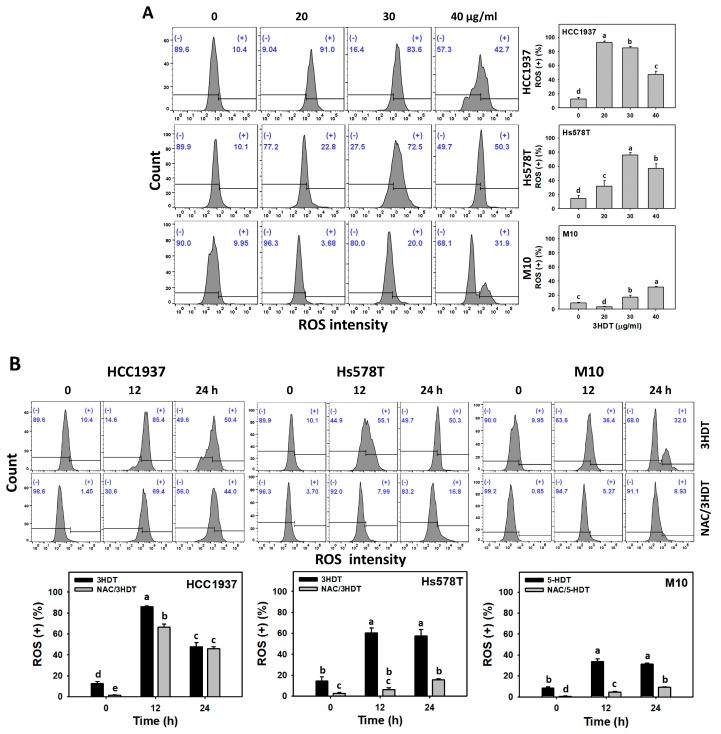
ROS status of 3HDT treatment. (**A**) ROS flow cytometry assay of 3HDT for 24 h. (**B**) ROS flow cytometry assay of NAC/3HDT. 3HDT and NAC/3HDT indicate 12 or 24 h 3HDT treatment (40 μg/mL) without and with NAC pretreatment, respectively. (+) is marked as ROS (+), which indicates the proportion with high ROS level, while (−) indicates the proportion with low ROS level. Data: mean ± SD (*n* = 3). Treatments differ significantly for multiple comparisons when the letters are not overlapping (*p* < 0.05).

ROS is a common indicator of cellular oxidative stress [29]. The fact that NAC reversed the 3HDT-induced antiproliferation warranted our study of the involvement of oxidative stress. Following the 3HDT treatment (0, 20, 30, and 40 μg/mL), the ROS (+) level was higher in TNBC cells than in normal cells (Figure 5A). Similarly, the 3HDT treatment (0, 12, and 24 h) showed a higher increase in ROS (+) levels for TNBC cells compared with normal M10 cells (Figure 5B). These results suggest that 3HDT promotes preferential ROS (oxidative stress) generation in TNBC cells but not normal cells.

Moreover, 3HDT showed more elevated ROS (+) levels compared with NAC/3HDT treatment of TNBC cells for 12 and 24 h (Figure 5B), suggesting that 3HDT promotes the preferential generation of ROS in TNBC cells via oxidative stress generation.

### 2.6. Mitochondrial Membrane Potential (MMP) Status of 3HDT-Treated TNBC Cells

**Figure 6:**
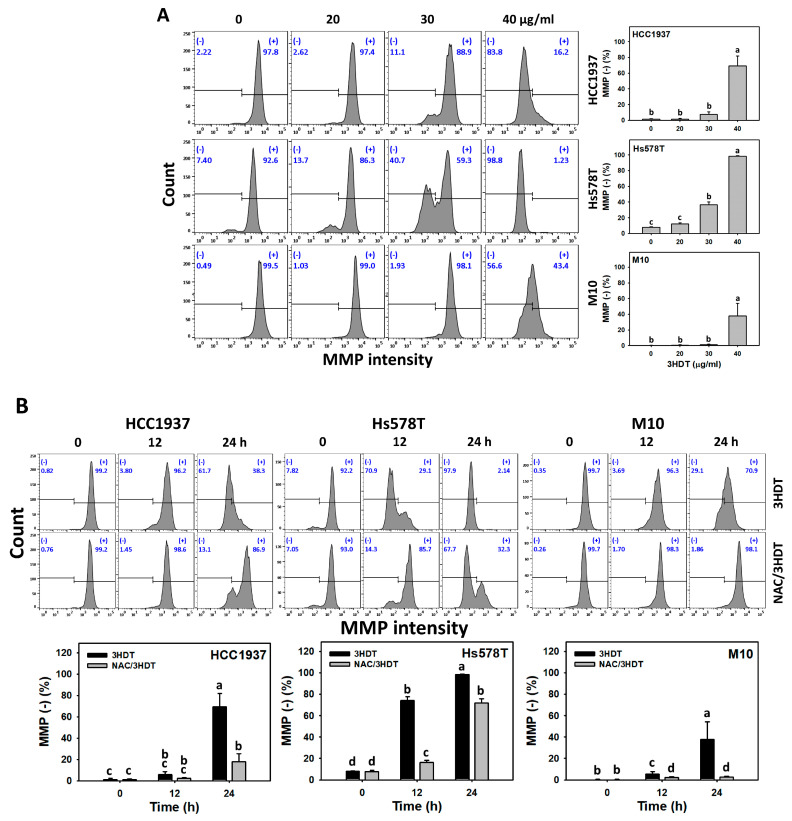
MMP status of 3HDT treatment. (**A**) MMP flow cytometry assay of 3HDT for 24 h. (**B**) MMP flow cytometry assay of NAC/3HDT. 3HDT and NAC/3HDT indicate 12 or 24 h 3HDT treatment (40 μg/mL) without and with NAC pretreatment, respectively. (−) is marked as MMP (−) that indicates the proportion with low MMP level, while (+) indicates the proportion with high MMP level. Data: mean ± SD (*n* = 3). Treatments differ significantly for multiple comparisons when the lower-case letters are not overlapping (*p* < 0.05).

In addition to ROS, another oxidative-stress-related indicator, namely MMP [30], was examined. Following 3HDT treatment, the MMP (−) level, i.e., MMP depletion status, was higher in TNBC cells in a dose-dependent manner than in normal cells (Figure 6A). Similarly, the 3HDT treatment showed more MMP (−) level increases in time-dependent manners for TNBC cells than normal M10 cells (Figure 6B). These results suggest that 3HDT promotes preferential MMP depletion (oxidative stress) in TNBC cells but not normal cells.

Moreover, 3HDT showed a higher MMP (−) level than NAC/3HDT treatment of TNBC cells (Figure 6B), suggesting that 3HDT promotes the preferential MMP depletion of TNBC cells via oxidative stress generation.

### 2.7. Glutathione (GSH) Status of 3HDT-Treated TNBC Cells

**Figure 7:**
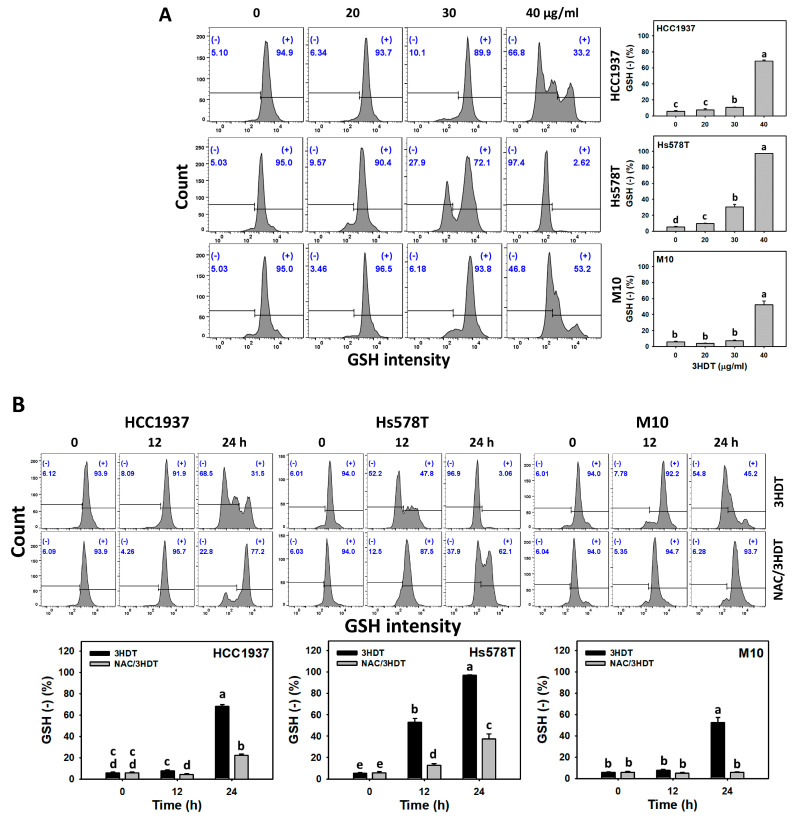
GSH status of 3HDT treatment. (**A**) GSH flow cytometry assay of 3HDT for 24 h. (**B**) GSH flow cytometry assay of NAC/3HDT. 3HDT and NAC/3HDT indicate 12 or 24 h 3HDT treatment (40 μg/mL) without and with NAC pretreatment, respectively. (−) is marked as GSH (−), which indicates the proportion with low GSH level, while (+) indicates the proportion with high GSH level. Data: mean ± SD (*n* = 3). Treatments differ significantly for multiple comparisons when the letters are not overlapping (*p* < 0.05).

GSH was examined to evaluate the change in cellular antioxidant levels [31] for monitoring oxidative stress [32]. Following the 3HDT treatment, the GSH (−) level, i.e., GSH depletion status, was higher in TNBC cells in a dose-dependent manner than in normal cells (Figure 7A). Similarly, the 3HDT treatment showed higher increases in the GSH (−) level of TNBC cells in time-dependent manners compared with normal M10 cells (Figure 7B). These results suggest that 3HDT promotes preferential GSH depletion (oxidative stress) in TNBC cells but not normal cells.

Moreover, 3HDT showed a more increased GSH (−) level than NAC/3HDT treatment of TNBC cells (Figure 7B), suggesting that 3HDT promotes the preferential GSH depletion of TNBC cells via oxidative stress generation. 

### 2.8. γH2A Histone Family Member X (γH2AX) and 8-Hydroxy-2-deoxyguanosine (8-OHdG) Status of 3HDT-Treated TNBC Cells

**Figure 8:**
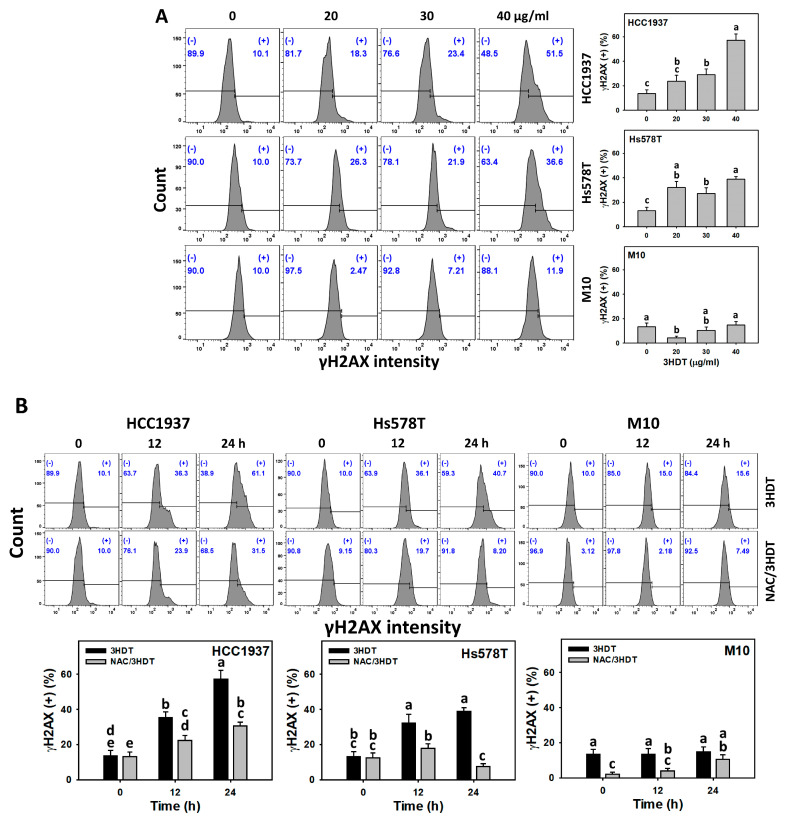
γH2AX status of 3HDT treatment. (**A**) γH2AX flow cytometry assay of 3HDT for 24 h. (**B**) γH2AX flow cytometry assay of NAC/3HDT. 3HDT and NAC/3HDT indicate 12 or 24 h 3HDT treatment (40 μg/mL) without and with NAC pretreatment, respectively. (+) is marked as γH2AX (+), which indicates the proportion with high γH2AX level, while (−) indicates the proportion with low γH2AX level. Data: mean ± SD (*n* = 3). Treatments differ significantly for multiple comparisons when the letters are not overlapping (*p* < 0.05).

**Figure 9:**
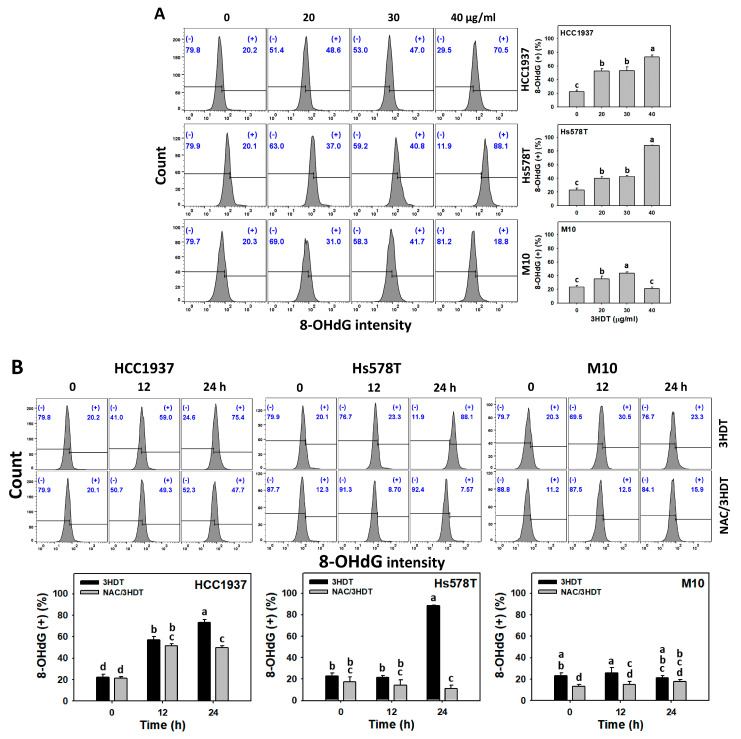
8-OHdG status of 3HDT treatment. (**A**) 8-OHdG flow cytometry assay of 3HDT for 24 h. (**B**) 8-OHdG flow cytometry assay of NAC/3HDT. 3HDT and NAC/3HDT indicate 12 or 24 h 3HDT treatment (40 μg/mL) without and with NAC pretreatment, respectively. (+) is marked as 8-OHdG (+), which indicates the proportion with high 8-OHdG level, while (−) indicates the proportion with low 8-OHdG level. Data: mean ± SD (*n* = 3). Treatments differ significantly for multiple comparisons when the letters are not overlapping (*p* < 0.05).

γH2AX [33] and 8-OHdG [34] were examined to evaluate the change in DNA damage levels for DNA double-strand breaks and oxidative DNA damage, respectively. Following 3HDT treatment (0, 20, 30, and 40 μg/mL), the γH2AX and 8-OHdG (+) levels were higher in TNBC cells than in normal cells (Figure 8A and Figure 9A). Similarly, the 3HDT treatment (0, 12, and 24 h) showed a higher increase in γH2AX and 8-OHdG (+) levels for TNBC cells compared with normal M10 cells (Figure 8B and Figure 9B). These results suggest that 3HDT promotes preferential DNA damage in TNBC cells but not normal cells.

Moreover, 3HDT showed higher γH2AX and 8-OHdG (+) levels in TNBC cells than NAC/3HDT treatment for 12 and 24 h (Figure 8B and Figure 9B), suggesting that 3HDT promotes the preferential generation of γH2AX and 8-OHdG (DNA damage) in TNBC cells via oxidative stress generation.

## 3. Discussion

The present investigation validated that 3HDT preferentially inhibited the proliferation of breast cancer cells but showed a low impact on normal cells. Several preferential antiproliferation mechanisms are discussed regarding breast cancer and normal cells.

Ginger extracts contain several natural products, such as gingerols, shogaols, gingediols, zingerone, dehydrozingerone, gingerinone, and diarylheptanoids [14,15,16]. Different ginger-derived compounds exhibit different sensitivities to breast cancer cells. For example, 1′ S-1′-acetoxyeugenol acetate showed an IC_50_ value of 14 μM in breast cancer cells (MCF 7) in a 36h MTT assay but kept 86.8% viability for normal breast cells [35]. 6-gingerol showed an IC_50_ value of 200 μM in breast cancer cells in a 48 h assay (MCF7 and MDA-MB-231) [36]. 6-shogaol showed an IC_50_ value of 23.3 μM in breast cancer cells (MCF7) in a 72 h sulforhodamine B assay [37]. Gingerenone A showed IC_50_ values of 61.4 and 76.1 μM in breast cancer cells MCF7 and MDA-MB-231, respectively, in a 48 h MTS assay [18].

The present study found that the IC_50_ values of 3HDT were 101.11 and 94.36 μM in breast cancer cells Hs578T and HCC1937, respectively, at 24 h in a CCK-8 assay, while normal breast cells (M10) kept the same viability of 3HDT as the control (Figure 1). In comparison, the IC_50_ values of cisplatin in MDA-MB-231 cells were undetectable within a 24 h MTT assay, i.e., 55% viability at 100 µM [38]. In contrast, the IC_50_ values of cisplatin were 32.38 and 66.42 µM for MDA-MB-468 [39] and MCF7 [40] cells, respectively, in a 24 h MTT assay. Therefore, 3HDT exhibits slightly lower sensitivity than cisplatin against TNBC cells. However, side effects may occur with cisplatin treatment [41].

Several natural products [42,43,44] were reported to inhibit cancer cell proliferation by generating oxidative stress [45]. Moreover, some natural products preferentially create more oxidative stress in cancer cells than in normal cells, leading to the preferential antiproliferation of cancer cells. For example, sinularin showed preferential antiproliferation, which is associated with preferential oxidative stress in breast cancer cells but not in normal cells [42], and fucoidan exhibited preferential antiproliferation and oxidative stress in oral cancer cells [46].

The present investigation showed similar results, namely the increased inhibition of 3HDT proliferation and enhanced oxidative stress generation in breast cancer cells compared with normal cells. The ROS and MMP levels were up- and downregulated in breast cancer cells but not in normal cells by 3HDT treatment, respectively (Figure 5 and Figure 6). Moreover, the ROS inhibitor NAC reverted the 3HDT-induced antiproliferation and oxidative stress generation. This finding validated that 3HDT-induced preferential antiproliferation was mediated by oxidative stress.

Antioxidant and oxidative stress have a reciprocal relationship [47,48]. Several anticancer reports demonstrated that antioxidant depletion was followed by ROS induction. For example, the nitrated [6,6,6]tricycle-derived compound SK1 triggered GSH depletion, leading to ROS overproduction in oral cancer cells [49]. Auranofin exhibits antiproliferation by ROS induction and GSH depletion in lung cancer cells [50]. Moreover, examining the status of antioxidant levels for 3HDT treatment is necessary because oxidative stress was upregulated. 3HDT also preferentially downregulated antioxidant levels, such as GSH, in breast cancer but not in normal cells (Figure 7). These results suggested that 3HDT-induced oxidative stress was partly attributed to GSH downregulation.

In addition to antiproliferation and antioxidant regulation, oxidative stress is commonly associated with apoptosis [46,51] and DNA damage [18,52,53,54]. For example, sinularin triggers preferential oxidative stress, leading to the preferential apoptosis and DNA damage of breast cancer cells but not normal cells [42]. Fucoidan shows preferential oxidative stress effects, contributing to the preferential apoptosis and DNA damage of oral cancer cells [46]. Similarly, in the present study, 3HDT induced more γH2AX and 8-OHdG levels of DNA damage as well as apoptosis (annexin V, caspase 3, caspase 8, and caspase 9) in breast cancer cells than in normal cells, which was reverted by NAC pretreatment. This finding validated that 3HDT-induced preferential DNA damage and apoptosis were associated with preferential oxidative stress in breast cancer cells rather than normal cells.

Some natural products show antiproliferative effects on both TNBC and non-TNBC cells. For example, the methanol extract of *Aaptos suberitoides* (MEAS), a marine-sponge-derived natural product, inhibits the cell viability of TNBC (HCC1937, MDA-MB-231, and MDA-MB-468) and non-TNBC (MCF7; ER+/PR+ type) cells [55]. Ginger-derived gingerenone A [18], *Physalis peruviana*-derived physapruin A [56], and *Sinularia flexibilis*-derived sinularin [42] suppress the proliferation for both TNBC (MDA-MB-231) and non-TNBC (SKBR3 (HER2+ type) and/or MCF7) cells. Moreover, some of these antibreast cancer agents also exhibit antiproliferative effects on other non-breast cancer cells. For example, physapruin A also suppresses oral cancer cell proliferation [57]. In addition to breast cancer cells, sinularin exerts antiproliferative function on renal [58] and oral [59] cancer cells. The typical characteristic of these drugs [18,55,56] is their ROS-inducing ability, which causes the inhibition of cancer cell proliferation. As 3HDT is also an ROS-inducing agent, the antiproliferative potential for non-TNBC and non-breast cancer cells warrants a full investigation in the future.

Although 3HDT induces lower apoptosis in normal (M10) cells compared with TNBC cells, the 3HDT-induced potential necrosis is higher in M10 cells than in TNBC cells. Moreover, 3HDT induces higher antiproliferative effects in TNBC cells than in M10 cells, suggesting that 3HDT-induced apoptosis causes higher antiproliferation than 3HDT-induced necrosis. This explanation is partly supported by the finding that ethyl acetate *Nepenthes* extract (EANT) shows necrosis at low concentrations and apoptosis at high concentrations, which causes low and high antiproliferation, respectively, in oral cancer cells [23]. Moreover, the necrosis determination of 3HDT treatment in the present study was based on the annexin V/7ADD method, which warrants a detailed future study.

Furthermore, more prolonged exposures to 3HDT may increase the sensitivity of breast cancer treatment. Combined treatment with clinical drugs or other natural products may also improve the effects of 3HDT. However, these suggestions still need further investigation in the future.

## 4. Materials and Methods

### 4.1. Plant Material, Extraction, and Isolation

3HDT was purified from the rhizomes of *Z. officinale*. The fresh rhizomes of *Z. officinale* (27.5 kg) were air-dried and repeatedly extracted with MeOH at room temperature for 48–72 h. The combined MeOH extracts were evaporated and partitioned to yield CH_2_Cl_2_ and H_2_O layers. The CH_2_Cl_2_ layer residue was separated into six fractions using column chromatography (CC) on Si gel with gradient systems of *n*-hexane/CH_2_Cl_2_/acetone *n*-hexane-acetone. Fraction 2 was purified using Si gel CC (*n*-hexane-acetone, 60:1) to yield 3HDT (*n*-hexane-acetone, 20:1, Rf = 0.43) [20]. The ^1^H NMR spectrum is provided in Supplementary Figure S1.

### 4.2. Chemical Profile of 3HDT

3HDT (MW = 378): yellow oil. [α]^25^_D_ + 4.63° (c 1.50, CH_2_Cl_2_). UV (CH_3_CN, λ_max_, nm): 230, 280. IR (ν_max_, cm^−1^): 3500, 1710, 1600, 1515. ^1^H NMR (500 MHz, CDCl_3_, δ, ppm, J/Hz): 0.88 (3H, t, J = 6.8, H-14), 1.27 (10H, m, H-9~H-13), 1.43 (2H, m, H-8), 2.20 (2H, q, J = 5.0, H-2), 2.52 (1H, dd, J = 7.2, 14.8, H-4a), 2.59 (1H, dd, J = 2.0, 14.8, H-4b), 2.78 (2H, t, J = 6.0, H-1), 3.92 (6H, s, 3′-OC*H*_3_ and 5′-OC*H*_3_), 4.01 (1H, m, H-3), 6.05 (1H, s, OH), 6.12 (1H, d, J = 16.0, H-6), 6.72 (2H, s, H-2′ and H-6′), 6.81 (1H, dt, J = 16.0, 5.6, H-7). ^13^C NMR (125 MHz, CDCl_3_, δ, ppm): 14.1 (C-14), 22.5 (C-13), 22.7 (C-12), 23.8 (C-11), 25.2 (C-10), 31.1 (C-1), 33.6 (C-9), 35.2 (C-8), 39.3 (C-2), 49.5 (C-4), 56.2 (3,5-OCH_3_), 71.3 (C-3), 107.7 (C-2′/C-6′), 125.6 (C-1′), 130.2 (C-6), 138.4 (C-4′), 147.3 (C-7), 151.1 (C-3′/C-5′), 199.9 (C=O). ESI-MS *m/z* 401 [M+Na]^+^. HR-ESI-MS *m/z* 401.2301 [M+Na]^+^ (calcd for C_22_H_34_O_5_Na, 401.2304) [20].

### 4.3. Chemicals

Inhibitors for oxidative stress, such as *N*-acetylcysteine (NAC) [60], were dissolved in 1X PBS and DMSO for drug pretreatments, i.e., 10 mM for 1 h. They were purchased from Sigma-Aldrich (St. Louis, MO, USA) and Selleckchem.com (Houston, TX, USA).

### 4.4. Cell Cultures and Viability

Two ATCC-derived human TNBC cell lines (HCC1937 and Hs 578T; Manassas, VA, USA) and one BCRC Cell Bank (HsinChu, Taiwan) normal breast cell line (H184B5F5/M10; M10) [61,62,63] were used. TNBC cells were incubated in RPMI (HCC1937) or DMEM/F12 (3:2) (Hs578T) medium, and M10 cells were maintained in alpha medium, supplemented by 10% bovine serum (Gibco, Grand Island, NY, USA) and P/S antibiotics [42]. According to user instructions, cell viability was assessed using CCK-8 assay (IMT Formosa New Materials, Kaohsiung, Taiwan) [21,22].

### 4.5. Cell Cycle Analysis

Cellular DNA within the fixed cells was detected using 7-aminoactinmycin D (7AAD; 1 μg/mL) for 30 min (Biotium; Hayward, CA, USA) [46]. The DNA signals were measured using a Guava easyCyte flow cytometer (Luminex, TX, USA).

### 4.6. Apoptosis Analysis

Annexin V/7AAD kit [46] (Strong Biotech; Taipei, Taiwan), caspase-Glo^®^ 3/7 [64], caspase-Glo^®^ 8, and caspase-Glo^®^ 9 [26] luminescence reagents (Promega; Madison, WI, USA) were applied to measure apoptosis, as described in the user’s manual. Annexin V/7AAD intensities were monitored using flow cytometry. Caspase 3/7, 8, and 9 activities were analyzed using a luminometer (Berthold Technologies GmbH & Co., Bad Wildbad, Germany). Moreover, apoptotic signaling, such as caspase 3, was examined following the user instructions. The activated caspase 3 was able to cleave the OncoImmunin’s specific peptides (Gaithersburg, MD, USA) to generate signaling for flow cytometry [65,66].

### 4.7. Analysis of ROS, MMP, and GSH

Oxidative stresses, including ROS, MMP, and GSH signals, were proportional to the fluorescence generated by their chemical reactions. These reactions were performed by adding 2′,7′-dichlorodihydrofluorescein diacetate (DCFH-DA; Sigma-Aldrich, St. Louis, MO, USA) (10 μM for 30 min), DiOC_2_(3) [42] (Invitrogen; San Diego, CA, USA) (5 nM for 30 min), and 5-chloromethylfluorescein diacetate (CMF-DA) (Thermo Fisher Scientific, Carlsbad, CA, USA) (5 μM for 20 min) [46] to cells. Finally, these signals were measured using flow cytometry.

### 4.8. DNA Damage Analysis (γH2AX and 8-OHdG)

DNA damage types of 75%-ethanol-fixed cells, such as γH2AX and 8-OHdG [46], were assessed using antibody detection followed by flow cytometry, as previously described.

### 4.9. Statistical Analysis

JMP 12 software (SAS Campus Drive, Cary, NC, USA) was employed to decide the significance of multiple comparisons. Data with different lower-case letters indicate significant results for three independent experiments. Data were presented as mean ± SD.

## 5. Conclusions

3HDT is a novel compound derived from *Z. officinale*; however, only chemical characterization was previously available. The present study first evaluated the antiproliferation effects of 3HDT using the example of TNBC breast cancer cells. 3HDT demonstrated preferential antiproliferation for TNBC cells compared with normal breast cells. The 3HDT-associated changes, such as apoptosis, caspase activation, oxidative stress, and DNA damage, were elevated in breast cancer cells compared with normal cells. NAC reverted these oxidative changes. In conclusion, 3HDT provides a promising anticancer agent with preferential antiproliferation, oxidative stress, apoptosis, and DNA damage effects on TNBC cells.

## Data Availability

Data are contained within the article.

## Supplementary Materials

The following supporting information can be downloaded at: https://www.mdpi.com/article/10.3390/ijms24065741/s1, Figure S1: The ^1^H NMR spectrum of 3HDT.
