# Supplementary figures and images for: Ginger-Derived 3HDT Exerts Antiproliferative Effects on Breast Cancer Cells by Apoptosis and DNA Damage
Source: Int J Mol Sci. 2023 Mar 17;24(6):5741. doi: 10.3390/ijms24065741 (PMC10054677; doi:10.3390/ijms24065741)

G9-11-2

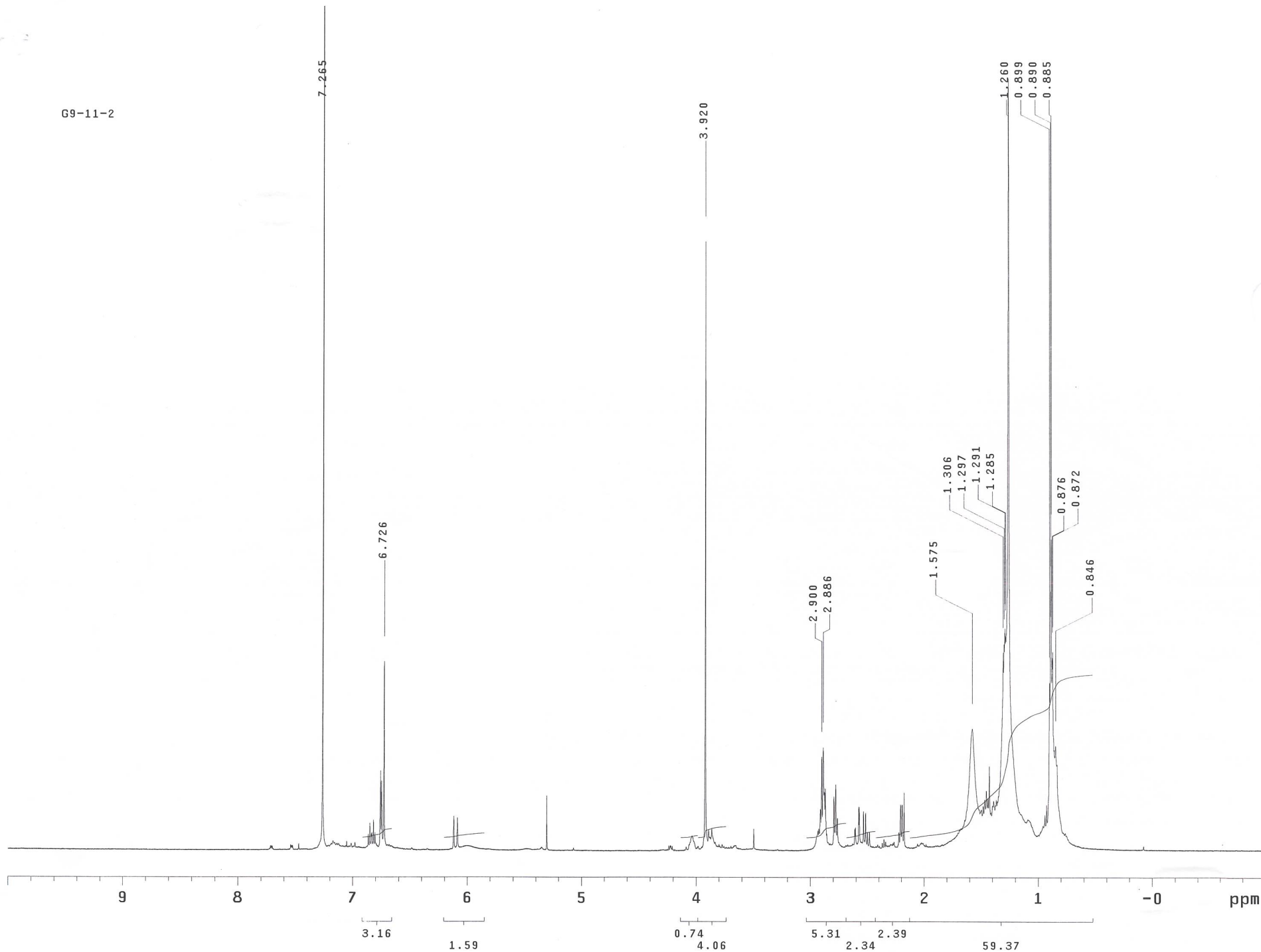

Supplement: Supplementary file 1 [file ijms-24-05741-s001.zip › ijms-2278317-supplementary.pdf]
